# The use of the NEDD8 inhibitor MLN4924 (Pevonedistat) in a cyclotherapy approach to protect wild-type p53 cells from MLN4924 induced toxicity

**DOI:** 10.1038/srep37775

**Published:** 2016-11-30

**Authors:** Lara J. Bou Malhab, Simon Descamps, Benedicte Delaval, Dimitris P. Xirodimas

**Affiliations:** 1 CRBM-CNRS, Cell Biology Research of Montpellier, UMR5237, Montpellier University, 1919 Route de Mende, 34293, Cedex 5, Montpellier, France

## Abstract

Targetting the ubiquitin pathway is an attractive strategy for cancer therapy. The inhibitor of the ubiquitin-like molecule NEDD8 pathway, MLN4924 (Pevonedistat) is in Phase II clinical trials. Protection of healthy cells from the induced toxicity of the treatment while preserving anticancer efficacy is a highly anticipated outcome in chemotherapy. Cyclotherapy was proposed as a promising approach to achieve this goal. We found that cytostatic activation of p53 protects cells against MLN4924-induced toxicity and importantly the effects are reversible. In contrast, cells with mutant or no p53 remain sensitive to NEDD8 inhibition. Using zebrafish embryos, we show that MLN4924-induced apoptosis is reduced upon pre-treatment with actinomycin D *in vivo*. Our studies show that the cellular effects of NEDD8 inhibition can be manipulated based on the p53 status and that NEDD8 inhibitors can be used in a p53-based cyclotherapy protocol to specifically target cancer cells devoid of wild type p53 function, while healthy cells will be protected from the induced toxicity.

Protein modification with the ubiquitin-like molecule (Ubl) NEDD8 (protein NEDDylation) is emerging as an important regulatory pathway^1^,^2^. With the exception of *S. cerevisiae*, the NEDD8 pathway is essential in all tested organisms^3^. NEDD8 has the highest sequence homology to ubiquitin amongst the family of Ubls but a distinct conjugation pathway exists. Covalent modification with NEDD8 occurs through the three key enzymatic activities E1, E2, E3 and is reversed by deconjugating (deNEDDylating) enzymes that remove NEDD8 from its targets. The well-established role of NEDD8 is to regulate protein degradation mediated by the family of Cullin-Ring-Ligases (CRL). Through covalent modification of cullins, NEDD8 stimulates the E3-ligase of CRLs promoting ubiquitination and proteasomal degradation of CRL targets, including proteins with established functions in cell cycle, DNA replication, DNA damage response and cell motility. Studies on non-cullin NEDD8 substrates identified additional roles of protein NEDDylation, including transcriptional activity and tumour suppressor function regulation, signalling, cell metabolism, apoptosis and histone modification^1^,^2^. Furthermore, global NEDDylation and expression of components of the NEDD8 machinery such as NAE and Ubc12 are found upregulated in lung adenocarcinoma, squamous-cell carcinomas and hepatocellular carcinoma^4^,^5^,^6^. Therefore, the NEDD8 pathway is implicated in the control of multiple cancer-related biological processes.

MLN4924 (Pevonedistat), a small molecule inhibitor of NEDDylation (specifically inhibits the NEDD8 E1-activating enzyme), has shown anti-tumour activities both *in vitro* and *in vivo*^7^. Treatment of tumour cells (breast, pancreatic, lung, ovarian, melanoma, B-cell like lymphomas) with MLN4924 induces apoptosis, and *in vivo* MLN4924 shows anti-tumour activity in mice harbouring human xenografts for solid and hematologic tumours^8^,^9^. These data suggested the NEDD8 pathway as a promising therapeutic target and MLN4924/Pevonedistat is in Phase II cancer clinical trials^10^, https://clinicaltrials.gov/ct2/show/NCT02610777?term=pevonedistat&rank=1).

Most chemotherapeutics show a preference in killing high-proliferating cells, a more common characteristic of cancer cells. Subsequently, chemotherapeutics display toxicity preference for cancer over healthy cells. Nevertheless, despite this relative selectivity, chemotherapeutics also induce toxicity in healthy cells accounting for the side effects of chemotherapy, which also constrains the doses that can be used. Therefore, chemotherapy is limited by its toxicity to normal cells^11^,^12^. A promising strategy called cyclotherapy, which aims at protecting normal tissues from chemotherapy side effects, has been developed. It is based on both the cell cycle and the status of the p53 tumour suppressor^12^,^13^,^14^,^15^,^16^. The p53 gene is one of the highest mutated/deleted genes in human cancers with over 50% of all cases containing mutations/deletions in the p53 locus, inactivating p53 function. The p53 pathway responds to a vast variety of stress signals and depending on the nature and strength of the applied stress (chemotherapeutics) p53 can induce cell cycle arrest (G1 and/or G2, cytostatic) or programmed cell death-apoptosis^17^,^18^.

Cyclotherapy relies on a two-step combination approach: A) Low-level cytostatic p53 activation, which will reversibly arrest healthy cells with wild-type p53 in G1/G2 phases of the cell cycle, while leaving cancer cells with mutant/deleted p53 cycling normally and B) Use of a chemotherapeutic that targets either S or M phase cells, which will specifically target the cycling cancer cells but not the arrested healthy cells^12^,^13^,^15^,^16^. Several cyclotherapy protocols have been successfully used, involving small molecule p53 activators, actinomycin D, leptomycin B, Nutlin-3, tenovin 6 at concentrations where they induce cytostatic p53 activation, in combination with S or M phase poisons, including vinblastine, vinorelbine, cytosine arabinoside, gemcitabine, polo-like and aurora kinase inhibitors, epitholones^15^. All above cyclotherapy protocols have been established *in vitro* and there is a sole validation of a cyclotherapy protocol *in vivo*, where Nutlin-3 protects mice against side effects induced by anticancer drugs^19^.

A profound cellular effect of inhibition of protein NEDDylation, which fulfills one of the criteria in cyclotherapy, is the accumulation of cells in S phase due to the continuous re-replication in the absence of mitosis. The phenotype is mainly attributed to the accumulation of the replication factor cdt1, an established cullin4A-DDB1 and/or cullin1-Skp2 based CRL substrate^1^,^20^. Cdt1 is required for replication firing but is degraded by CRLs, in S phase (cullin4A-DDB1) or throughout the cell cycle (cullin1-Skp2) to prevent re-replication^20^.

In this study, we demonstrate the use of MLN4924 in a p53-based cyclotherapy protocol. We found that pre-treatment of wild type p53 cells with low doses of actinomycin D (LDActD) or Nutlin-3 completely protects these cells against the cytotoxic effects of MLN4924. In contrast cells with no or mutant p53 remain highly sensitive to MLN4924. Importantly, the cytostatic effects on wild type p53 cells are fully reversible upon drug removal. Using zebrafish as *in vivo* model system we further show that pre-treatment with actinomycin D also protects embryos from MLN4924 induced apoptosis. The studies show that the effects of NEDD8 inhibition can be manipulated by a p53-dependent cell cycle arrest and provide a protocol for the use of NEDD8 inhibitors in p53-based cyclotherapy in the clinic. It also indicates that the effectiveness of MLN4924/Pevonedistat used in combination therapies may depend on the patient’s p53 status.

## Results

### Low doses of actinomycin D specifically protect wild type p53 cells from MLN4924-induced toxicity

Actinomycin D (Dactinomycin) is one of the oldest chemotherapy drugs, and a well-established p53 activator, which has been used in the treatment of a variety of cancers^21^,^22^. In cyclotherapy, the protective pre-treatment drugs are used at concentrations and duration that neither affect the cycling of cancer cells nor cause toxicity to normal cells. At low doses of actinomycin D (1–4 nM, LDActD), p53 activation causes a cytostatic effect through G1/G2 cell cycle arrest^15^. For our cyclotherapy approach, we used isogenic HCT116 colorectal cancer cell lines, HCT116^+/+^p53 with wild type p53 or HCT116^−/−^p53, which do not express full-length p53 but some of the identified p53 isoforms^23^.

**Figure 1:**
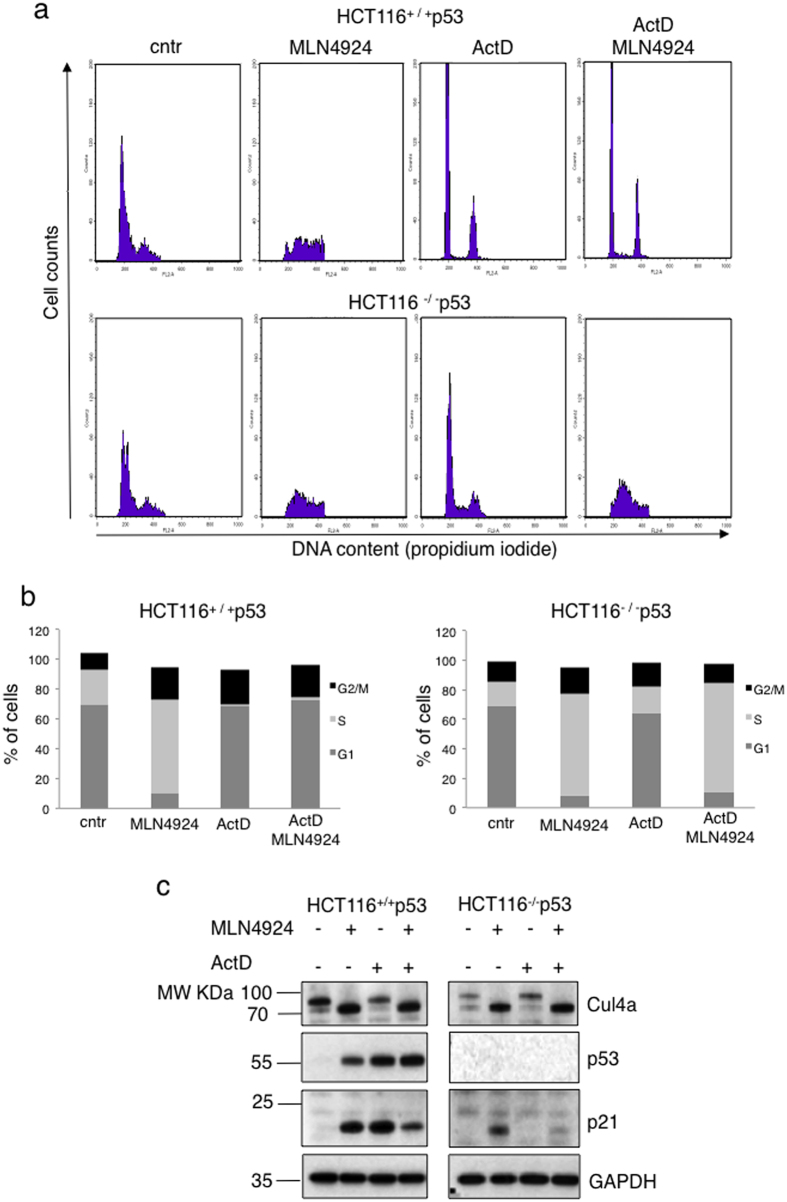
Pre-treatment with LDActD protects HCT116^+/+^p53 but not HCT116^−/−^p53 cells from MLN4924-induced toxicity. (**a**) HCT116^+/+^p53 and HCT116^−/−^p53 were pre-treated with ActD (2.5 nM) for 24 hrs before MLN4924 (1 μM) was added. Cells were harvested 24 hrs later and used for cell cycle analysis. (**b**) The average values of 4 independent experiments in (**a**) are presented as % of cells in G1, S, G2/M. (**c**) Western blot analysis for the indicated proteins from experiment performed in (**a**) (cropped blots). GAPDH was used as loading control.

**Figure 2:**
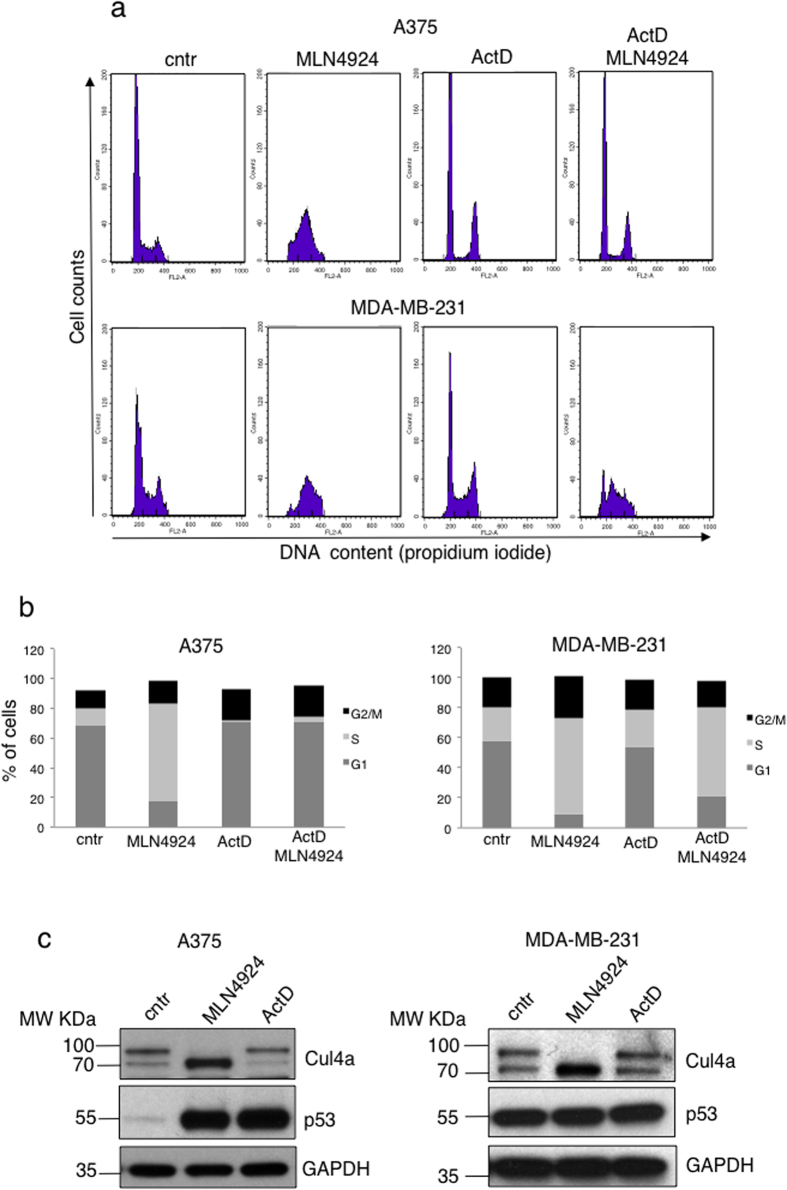
Pre-treatment with LDActD protects wild type p53 A375 cells but not mutant p53 MDA-MB-231 cells from MLN4924-induced toxicity. (**a**) Similar experiment as in Fig. 1 was performed in A375 (wild type p53) and MDA-MB-231 (mutant p53) cells. (**b**) The average values of 3 independent experiments in (**a**) are presented as % of cells in G1, S, G2/M. (**c**) Western blot analysis for the indicated proteins from the experiment performed in (**a**) (cropped blots). GAPDH was used as loading control.

Cells were treated either with LDActD (2.5 nM) or with MLN4924 (1 μM) or pre-treated with LDActD for 24 hrs followed by MLN4924 treatment. By FACS analysis we determined that LDActD causes a profound cell cycle arrest at G1 and G2 in HCT116^+/+^p53 but has minimal effects on HCT116^−/−^p53 cells (Fig. 1a,b). In contrast, at the used doses MLN4924 causes a dramatic S phase arrest in both cell lines (Fig. 1a,b). In the cyclotherapy regime, LDActD pre-treatment for 24 hrs, followed by MLN4924 treatment, resulted in a drastic S phase arrest in HCT116^−/−^p53 similarly to MLN4924 single treatment (Fig. 1a,b). However, MLN4924 had minimal effects on the cell cycle of the LDActD pre-treated HCT116^+/+^p53 cells, which remained arrested at G1/G2 phase, therefore protected from the cytotoxic effects of MLN4924 (Fig. 1a,b). By western blot analysis we determined that under the above conditions LDActD activated p53 with no effect on cullin-NEDDylation, whereas MLN4924 completely blocked cullin-NEDDylation and caused accumulation of the well-established CRL target p21 (Fig. 1c). Therefore, p53 dependent G1/G2 cell cycle arrest can protect cells against the dramatic S phase arrest induced by MLN4924 despite the inhibition of CRL activity. Similar effects were observed when comparing A375 melanoma cancer cells with wild type p53 and MDA-MB-231 breast cancer cells with mutant p53 (Fig. 2a,b,c). When both compounds were added simultaneously in an asynchronous cell population a combination of the cell cycle effects of the compounds when used alone was observed, suggesting that the compounds are not synergistic (data not shown).

**Figure 3:**
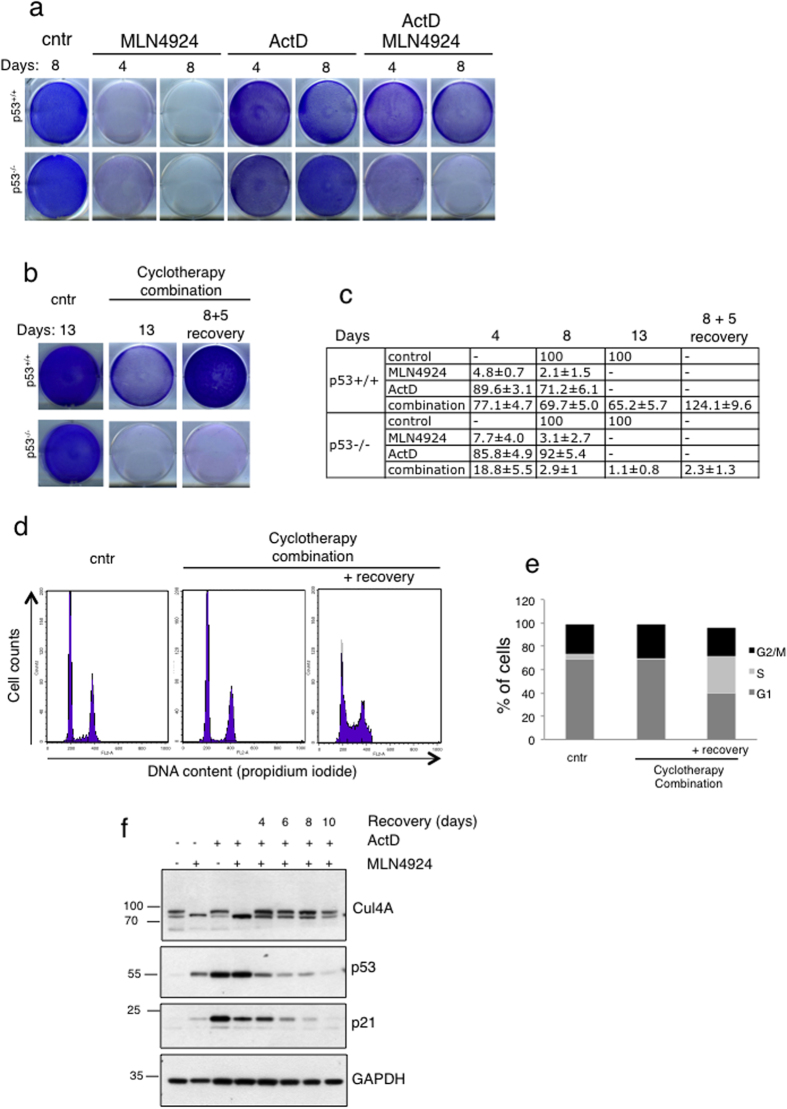
The induced cytostatic effects on wild type p53 cells are reversible. (**a**) Cells were seeded in 6-well dishes and treated as indicated (2.5 nM ActD, 1 μM MLN4924) before colonies were fixed with methanol and stained with Giemsa. (**b**) HCT116^+/+^p53 and HCT116^−/−^p53 cells were seeded in 6-well dishes and treated or not (cntr) with the LDActD/MLN4924 combination. After 8 days of treatment (conditions where the HCT116^−/−^p53 cells were completely eliminated) drugs were removed and cells were allowed to recover in drug-free medium or the cyclotherapy combination was maintained for 5 more days. Clonogenic assay was performed as described in (**a**). (**c**) Plates from (**a**) and (**b**) were scanned and signals were quantified with Image Gauge. The average values from 3 independent experiments are presented as % difference to the control cells of 8 (a) and 13 (b) days, +/− S.D. (**d**) HCT116^+/+^p53 cells were treated with LDActD for 24 hrs before MLN4924 was added for another 48 hrs. Drugs were then removed and cells allowed to recover in drug-free medium for 48 hrs and used for cell cycle analysis. (**e**) The average values from 3 replicate experiments in (**d**) are presented as % of cells in G1, S, G2/M. (**f**) Similar experiment as in (**d**). Cells allowed to recover for the indicated periods in drug-free medium before cell extracts were analysed for the indicated proteins by western blotting (cropped blots).

Based on the FACS analysis we then performed clonogenic assays to determine cell survival under the above cyclotherapy protocol. At the used cell density, LDActD had minimal effect on survival both in HCT116^+/+^p53 and HCT116^−/−^p53 cells (Fig. 3a,c). As expected, treatment with MLN4924 alone dramatically reduced survival for both cell lines (Fig. 3a,c). However, the pre-treatment with LDActD, clearly protected HCT116^+/+^p53 cells from the MLN4924 induced toxicity but not HCT116^−/−^p53 cells (Fig. 3a,c). One of the critical aspects in cyclotherapy is to determine whether the induced cytostatic effects in wild type p53 cells are reversible. We followed the LDActD-MLN4924 cyclotherapy protocol in HCT116^+/+^p53 and HCT116^−/−^p53 for a period of 8 days. Under these conditions there was a clear difference in cell survival between HCT116^+/+^p53 and HCT116^−/−^p53 cells (Fig. 3a,c). The HCT116^+/+^p53 cells arrested at G1/G2 phase upon the cyclotherapy treatment can increase in cell number upon release in medium with no drugs (Fig. 3b,c). No recovery was detected for the treated HCT116^−/−^p53 cells, indicating that the used cyclotherapy protocol eliminated all p53 null cells (Fig. 3b,c). Similar effects were observed by cell cycle analysis, where arrested HCT116^+/+^p53 cells can re-enter the cell cycle during the recovery period, evident by an increase of cells in S phase (Fig. 3d,e). Importantly, recovery in HCT116^+/+^p53 was also observed at the molecular level, as the levels of p53, p21 and cullin-NEDDylation recovered to the level of untreated cells (Fig. 3f). Therefore, the LDActD-MLN4924 combination fulfils all key criteria for a p53-based cyclotherapy approach.

### The LDActD pre-treatment reduces MLN4924-induced apoptosis in zebrafish embryos

**Figure 4:**
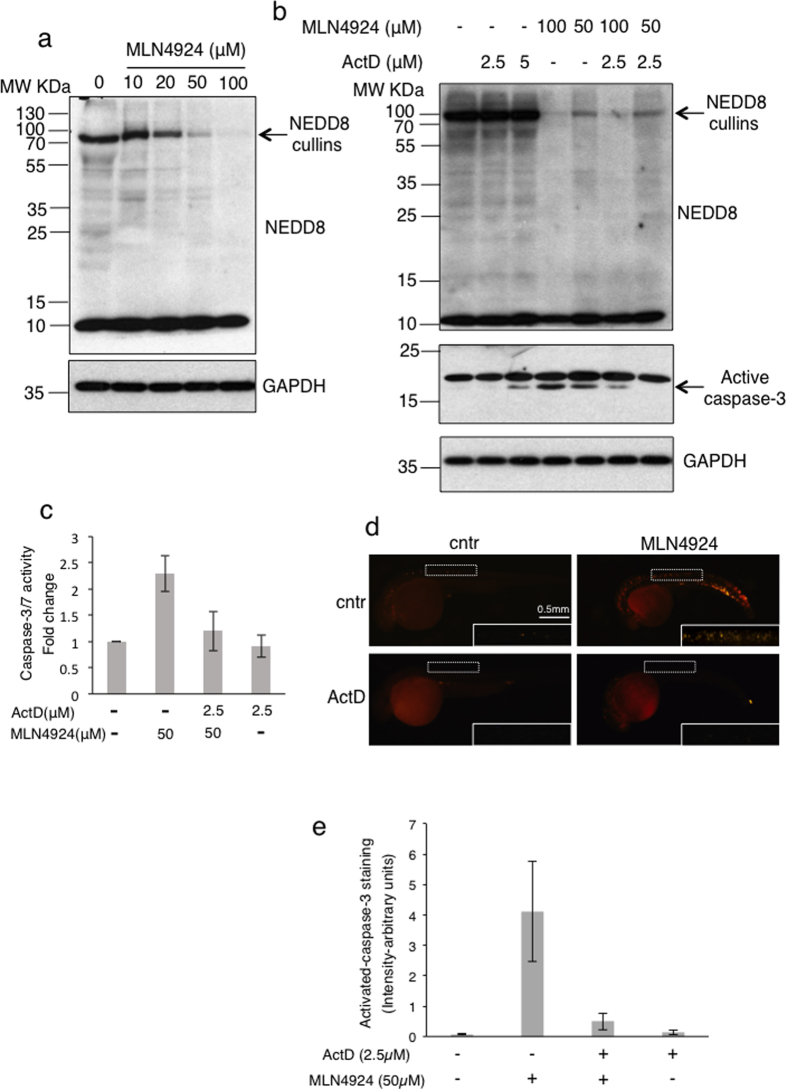
Pre-treatment with ActD protects zebrafish embryos from MLN4924-induced toxicity. (**a**) Zebrafish embryos were treated with the indicated doses of MLN4924 for 24 hrs before cell extracts were used for western blot analysis (cropped blot for GAPDH). (**b**) Similar experiment as in (**a**) except embryos were pre-treated with the indicated doses of ActD 4 hrs before MLN4924 addition. Extracts were analysed by western blotting for the indicated proteins (cropped blots for caspase-3, GAPDH). (**c**) Similar experiment as in (**b**) and extracts were used to measure cell viability and caspase-3/7 activity. Values represent the average of 3 independent experiments as fold change in caspase-3/7 activity normalised to cell viability, +/−S.D. as error bars. (**d**) Zebrafish embryos were treated as in (**b**) and used for immunofluorescence analysis with active caspase-3 antibody. Selected areas (white boxes-enlarged insets) were used to quantify the active caspase-3 signal, represented in (**e**) as arbitrary units of intensity, n = 25, error bars +/−S.D.

An important issue in cyclotherapy is to determine the effectiveness of the protocol in reducing the *in vivo* toxicity in healthy cells of the tested compound. Zebrafish has proven a powerful model system to test compounds toxicity, based on its high gene homology (>80%) and physiological similarities with humans. Numerous studies also confirmed that zebrafish can serve as an intermediate step between cell-based evaluation and conventional animal testing^24^. As the NEDD8 pathway is essential for development we hypothesized that zebrafish embryos would be a good system to assess the toxicity induced by inhibition of NEDDylation by MLN4924 and test whether toxicity could be reduced by pre-treatment with ActD. We first established the MLN4924 concentration required for an effective inhibition of NEDDylation *in vivo* in zebrafish embryos. 24 hours post fertilization (hpf) embryos were treated for 24 hrs with different concentrations of MLN4924 and extracts were analyzed by western blotting. We found that a dose of 50 μM was required to produce a significant inhibition of the NEDD8 pathway including cullin-NEDDylation (Fig. 4a). Under these conditions cell extracts were used to assess the activation of caspase-3 by western blotting or to measure caspase-3/7 activity, indicative of an apoptotic response. Inhibition of NEDDylation increased apoptosis (Fig. 4b,c), consistent with induced toxicity of MLN4924 in zebrafish embryos. We then tested whether pre-treatment of embryos with ActD could protect them from the MLN4924 induced apoptosis. We performed a titration of ActD and found that 2.5 μM did not produce any toxicity in the embryos when used alone, judged by morphology and caspase-3/7 activity (Fig. 4b,c). Importantly, MLN4924-induced apoptosis was reduced to almost background levels upon pre-treatment with ActD (Fig. 4b,c, ActD-2.5 μM/MLN4924–50 μM combination). In agreement with these results, whole mount immunofluorescence for active caspase-3 showed increased apoptosis upon MLN4924 treatment, which was rescued by pre-treatment with ActD (Fig. 4d,e). The data are consistent with the *in vitro* studies, indicating that MLN4924-induced apoptosis in healthy tissues can be rescued by ActD pre-treatment.

## Discussion

The ubiquitin pathway is regarded as a highly promising target for therapeutic intervention. The success of the inhibitor of the proteasome bortezomib in the clinic for the treatment of multiple myeloma, provided the proof of principle that blocking protein degradation has a high therapeutic potential^25^. The inhibitor of the NEDD8 pathway MLN4924 fulfils this criterion, as it is estimated that the inactivation of CRLs blocks the degradation of 20% of proteins targeted by the proteasome pathway^7^,^26^. MLN4924 has shown promising anti-tumour activity both *in vitro* and *in vivo* for haematological and solid tumours when used as single agent. Several *in vitro* studies indicated a synergistic anti-tumour activity for the combination of MLN4924 with ionizing radiation, DNA alkylating and alkylating-like chemotherapeutics^1^. A Phase II clinical trial for the combination of MLN4924 with the nucleoside analogue Azacitidine in patients with acute myeloid leukemia and myelodysplastic syndromes has been initiated^10^ https://clinicaltrials.gov/ct2/show/NCT02610777?term=pevonedistat&rank=1). The synergy of combined chemotherapeutics in inducing anti-tumour activity is a desired outcome. An exciting aspect in cyclotherapy is that the used combinations aim to selectively destroy cancer cells with mutant or no p53 and to protect healthy cells against the side effects of the used chemotherapy. This also allows increasing the maximum tolerated dose for the used S/M phase targeting compound.

In this study, we show that the LDActD-MLN4924 combination fulfils all the criteria to be used in a p53-based cyclotherapy approach. Pre-treatment with LDActD fully protected wild type p53 cells against the cytotoxic effects of MLN4924 even at high doses (1 μM), which when used alone produce a dramatic S phase arrest within 24 hrs of treatment. This also indicates that S phase entry is an absolute requirement for MLN4924 to induce cytotoxicity. While our studies focussed on the use of ActD as it is a drug commonly used for the treatment of a variety of cancers, we found that Nutlin-3, another p53 activator which has been successfully used in cyclotherapy, could protect HCT116^+/+^p53 but not HCT116^−/−^p53 cells from MLN4924-induced toxicity with similar efficiency to ActD (supplementary information, Fig. S1). Therefore, MLN4924 could be combined in cyclotherapy approaches with multiple and more specific p53 activators.

Activation of the p53 pathway is generally regarded as a desired effect of chemotherapy to induce cell death in cancer cells with wild-type p53. In principle, inhibition of NEDDylation by MLN4924 satisfies this requirement as it activates the p53 pathway through several proposed mechanisms, including the inactivation of CRL function, deNEDDylation of ribosomal proteins and p53 itself^1^,^27^. However, recent studies indicated that the MLN4924-induced p53 activation may instead protect cells against MLN4924, as either p53 null cells or wild type p53 cells where p53 was knockdown have increased sensitivity to MLN4924 compared to wild type p53 or control cells respectively^27^,^28^. Our data show that entry of cells in S phase is an absolute requirement for MLN4924 to induce toxicity. We propose that within an unsynchronised cell population, the p53 activation induced by MLN4924 will cause a G1/G2 arrest for a proportion of cells, preventing their entry into S phase and rescuing them from MLN4924-induced toxicity. This hypothesis is supported by the presented studies, where prior arrest of the whole cell population at G1/G2 by ActD/Nutlin-3, fully protects cells from MLN4924. Therefore, the MLN4924-induced p53 activation is cytoprotective, providing an explanation why cells devoid of wild type p53 activity are more sensitive to NEDD8 inhibition, especially at low doses^27^,^28^. This also raises the possibility of combining MLN4924 with inhibitors of the p53 pathway such as pifithrin, which should sensitise tumours with wild type p53 to the cytotoxic effects of MLN4924^1^,^29^.

The NEDD8 pathway is essential for development and treatment of zebrafish embryos with MLN4924 induces apoptosis. The rescue of the apoptotic effects by pre-treatment of embryos with ActD provides a proof of principle that such combination protocol may reduce side effects by MLN4924 *in vivo*. It is also consistent with the idea that cells with reduced rates of proliferation are less sensitive to NEDD8 inhibition. While MLN4924 is generally well-tolerated, common and MLN4924-specific side effects have been observed in clinical trials^10^.

The current studies show that MLN4924 can be used in a p53 based cyclotherapy protocol, which could help reducing MLN4924 cytoxicity in healthy cells while preserving and possibly augmenting MLN4924 anti-tumour activity in cancer cells with mutant or no p53. The studies also provide the proof of principle for testing NEDD8 inhibitors in combination therapies based on the patient’s p53 status.

## Methods

### Chemicals

Commonly used chemicals were of analytical grade, supplied by VWR and Sigma-Aldrich. MLN4924 was provided by Millennium Pharmaceuticals, doxorubicin from Enzo Life Sciences, actinomycin D from Sigma Aldrich and Nutlin-3 from Calbiochem. Trypsin was purchased from Promega.

### Antibodies

Mouse anti-p53 DO-1 antibody (in house), rabbit anti-cullin4A (Abcam, ab34897), rabbit anti-p21 (C-19) and mouse anti-GAPDH (sc-47724) from Santa Cruz Biotechnology, rabbit anti-NEDD8 (Gene Tex, Y297), anti-activated caspase-3 antibody for whole mount IF for zebrafish and western blotting (BD Biosciences 559565).

### Tissue culture and cellular assays

Isogenic HCT116 colorectal carcinoma, A375 human melanomas, MDA-MB-231 breast cancer cells were cultured in Dulbecco’s Modified Eagle’s Medium (DMEM, Gibco) with 50 U/ml Penicillin, 50 μg/ml Streptomycin and 10% FCS (fetal calf serum) at 37 °C, 5% CO2.

### SDS-polyacrylamide gel electrophoresis and western blotting

Proteins were resolved in NuPage 12% or 4–12% Bis-Tris precast gels (Invitrogen) before transferred onto PVDF membrane (Millipore). Membranes were blocked with 5% milk solution (PBS with 5% skimmed milk and 0.1% Tween-20, boiled and filtered) for 1 hr at room temperature. Primary antibodies diluted in PBS 0.1% Tween-20 with 3% BSA and 0.1% NaN_3_ were incubated with the membranes overnight at 4 °C. Membranes were washed 2x with PBS 0.1% Tween-20 (10 min each) prior to the incubation of the corresponding secondary antibodies (Sigma-Aldrich), which were diluted 1:2000 in 5% milk solution, at room temperature for 1 hr. Membranes were washed 4x with PBS 0.1% Tween-20 (10 min each) followed by 2x with PBS (5 min each). Detection was performed with ECL Western Blotting Detection Reagents (Amersham) and exposed to Medical Film (Konica Minolta).

### Cell cycle analysis

Fluorescence Activated Cell Sorting (FACS) was used to analyse the cell-cycle distribution. Cells were seeded in 6-well plates at required density (1 × 10^5^/well). ActD (2.5 nM)/Nutlin-3 (10 μM) and MLN4924 (1 μM) were added 24 and 48 hrs post-seeding respectively. Cells were harvested using trypsination, spun down and resuspended in 1 ml of PBS. Cells were then fixed in 70% ethanol and resuspended in 1 ml PBS, containing RNAse A (10 μg/ml, Invitrogen) propidium iodide (20μg/ml, Sigma, P4170) and incubated for 30 min at room temperature. Samples were analysed using a Becton Dickinson FACScan operated by the CELLQuest software.

### Giemsa staining

Cells were seeded in 6-well plates at the required density (1 × 10^5^/well) and treated as indicated. Cells were fixed with methanol for 7 min after which methanol was removed and plates left to dry. Cells were stained with 5% Giemsa (Sigma-Aldrich) for at least 1 hr before being washed with distilled water to remove excess stain. Plates were left to dry and were scanned. Images were quantified by Image Gauge and values are presented as the average of 3 independent experiments +/− standard deviation (S.D.).

### Zebrafish

Zebrafish (Danio rerio) AB lines were raised and used according to standard laboratory protocols^30^. Zebrafish use was performed in agreement with European Union guidelines for the handling of laboratory animals (http://ec.europa.eu/environment/chemicals/lab_animals/home_en.htm ) and was approved by the Direction Sanitaire et Vétérinaire de l’Hérault and Comité d’Ethique pour l’Expérimentation Animale under reference B-34-172-39.

Sanitaire et Vétérinaire de l’Hérault and Comité d’Ethique pour l’Expérimentation Animale under reference B-34-172-39. Fertilized eggs were kept at 28 °C in E3 solution with or without 0.003% 1-phenyl-2-thiourea (PTU, Sigma-Aldrich) to suppress pigmentation and staged according to hours post fertilization or hpf^30^. 6 hpf embryos were transferred to a 24-well plate (25 embryos/well) for drug treatments, diluted in E3 media +1% DMSO. 24 hpf embryos were pre-treated either with control (ethanol+DMSO) or ActD for 4 hrs before MLN4924 was added for further 24 hrs incubation. 1% DMSO was added in each condition. Dechorionated embryos were either transferred to deyolking solution (ice cold Ringer’s solution +0.3 mM PMSF +1 mM EDTA), passed several times through a 200 μl pipet tip to remove their yolk then rinsed in ice cold ringer and used for protein extraction (see below) for western blotting and caspase-3/7 activity assays (48 hpf) or fixed for immunofluorescence (30 hpf, n = 25 embryos per condition from 3 independent experiments) as previously described^31^ and imaged using a AxioZoom V16 microscope controlled by Zen software (Zeiss). The Integrated Density tool in Image J was used to measure the signal in the indicated areas (then expressed as fluorescence intensity in arbitrary units).

### Protein extracts from zebrafish embryos and apoptosis measurement

After treatment with ActD and/or MLN4924, 15 embryos were manually dechorionated, washed with ice-cold PBS 1x and after yolk removal were resuspended in cell lysis buffer (1 mM EDTA, 10% glycerol, 0.7% NP40, 1 mM DTT, 20 mM HEPES, pH8 and 280 mM KCl). Lysis was performed by incubating samples on ice for 10 min before centrifuged at 14000 rpm for 30 min at 4 °C^32^. Protein concentration of cleared lysates was measured with the BCA reagent (Pierce) and equal amount of total protein was used for gel electrophoresis and western blots. Extracts were also used for measuring caspase-3/7 activity and cell viability with the Caspase-Glo and CellTiter-Glo Promega kits respectively according to manufacture’s instructions in a 96-well plate. Caspase-3/7 values were normalised against cell viability and presented as fold change. Values represent the average of 3 independent experiments and error bars as S.D.

## Additional Information

**How to cite this article**: Malhab, L. J. B. *et al*. The use of the NEDD8 inhibitor MLN4924 (Pevonedistat) in a cyclotherapy approach to protect wild-type p53 cells from MLN4924 induced toxicity. *Sci. Rep.* **6**, 37775; doi: 10.1038/srep37775 (2016).

**Publisher's note:** Springer Nature remains neutral with regard to jurisdictional claims in published maps and institutional affiliations.

## Supplementary Material

Supplementary Information
