# Supplementary material for: The use of the NEDD8 inhibitor MLN4924 (Pevonedistat) in a cyclotherapy approach to protect wild-type p53 cells from MLN4924 induced toxicity
Source: Sci Rep. 2016 Nov 30;6:37775. doi: 10.1038/srep37775 (PMC5129021; doi:10.1038/srep37775)

The use of the NEDD8 inhibitor MLN4924 (Pevonedistat) in a cyclotherapy approach to protect wild-type p53 cells from MLN4924 induced toxicity.

Lara J. Bou Malhab, Simon Descamps, Benedicte Delaval and Dimitris P. Xirodimas

Supplementary Information

S1

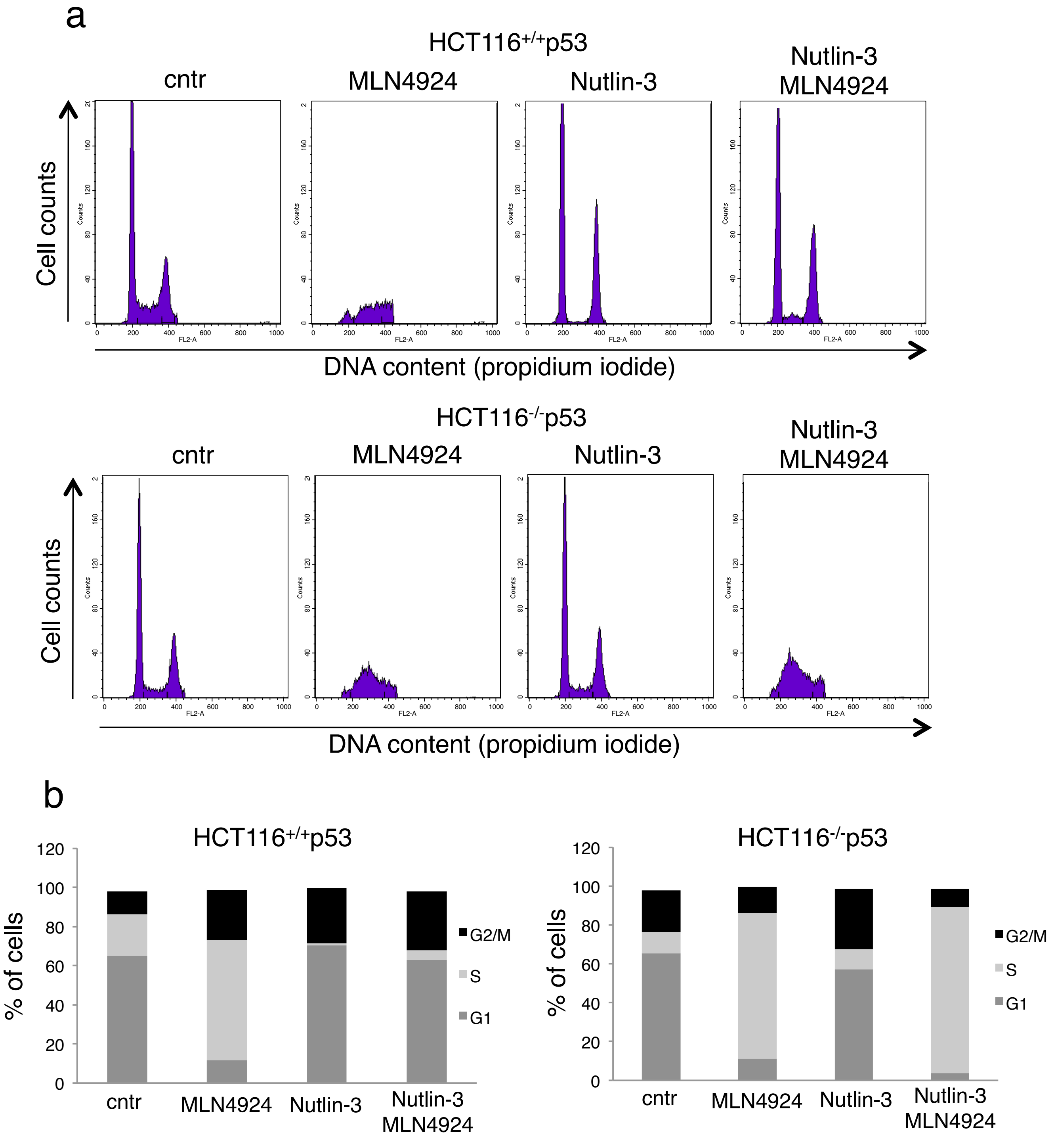

Pre-treatment with Nutlin-3 protects HCT116$^{+/+}$p53 but not HCT116$^{-/-}$p53 cells from MLN4924-induced toxicity.
a) HCT116$^{+/+}$p53 and HCT116$^{-/-}$p53 cells were treated and analysed by FACS as described in Fig. 1, except that Nutlin-3 (10μM) was used instead of ActD. b) The average values of 3 replicate experiments are presented as % of cells in G1, S, G2/M.

Blots

Figure 1B

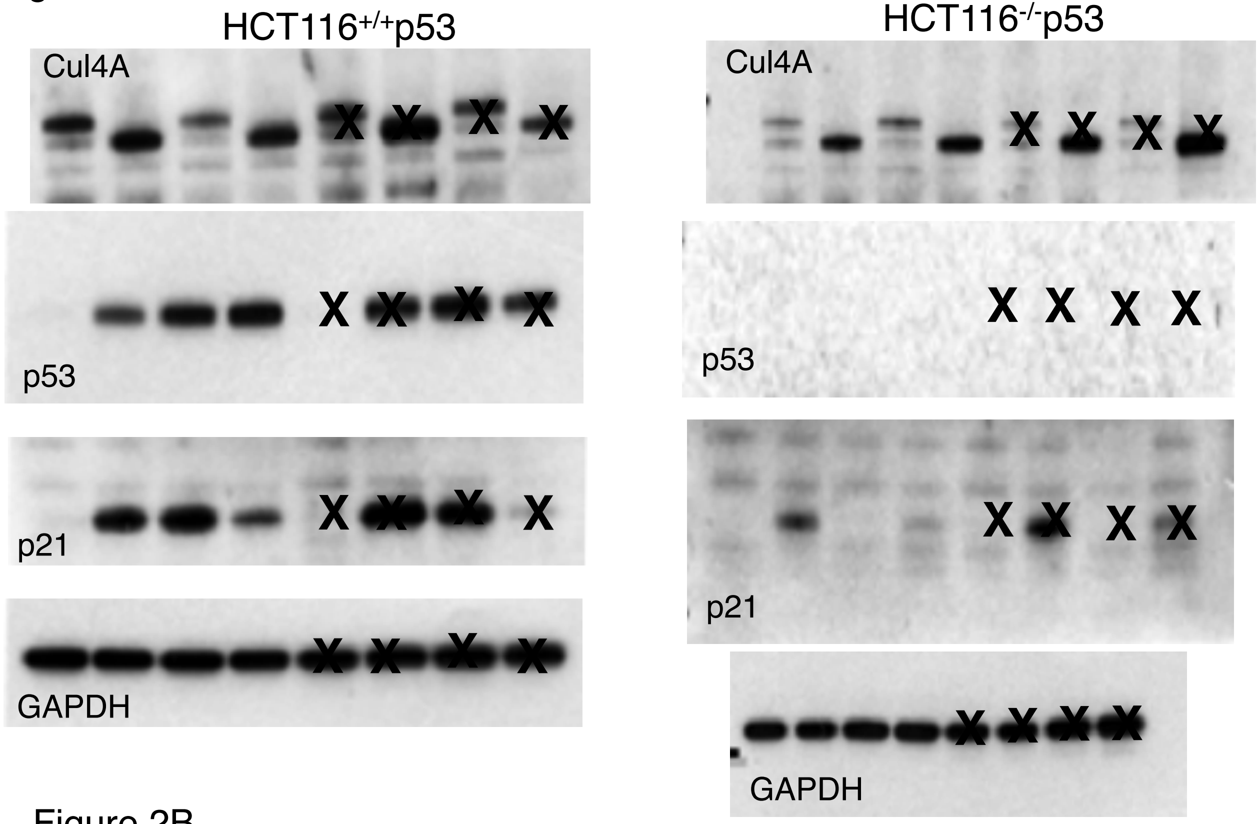

Figure 2B

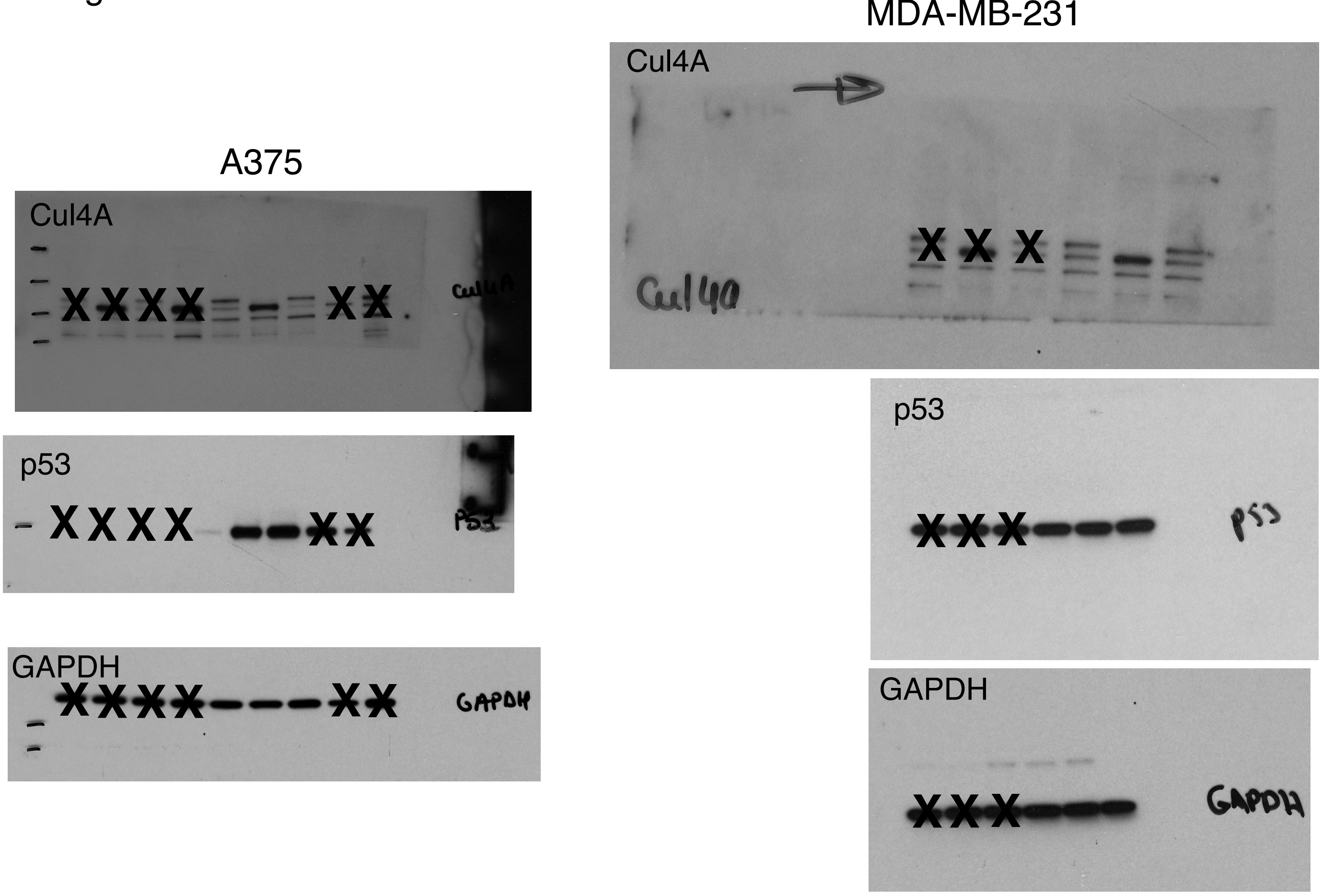

Figure 3D

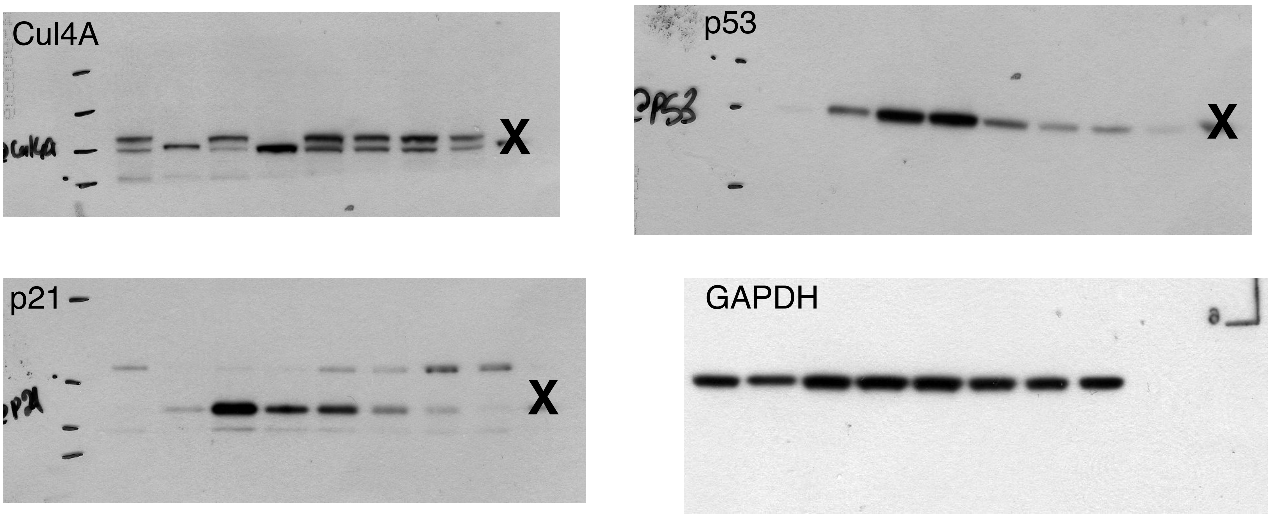

Figure 4B

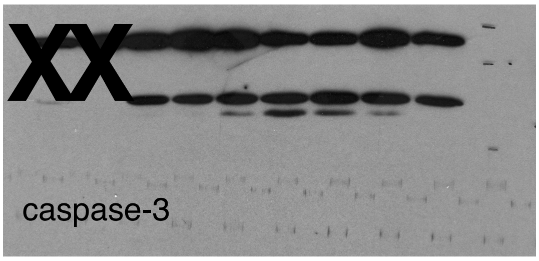

Figure 4A

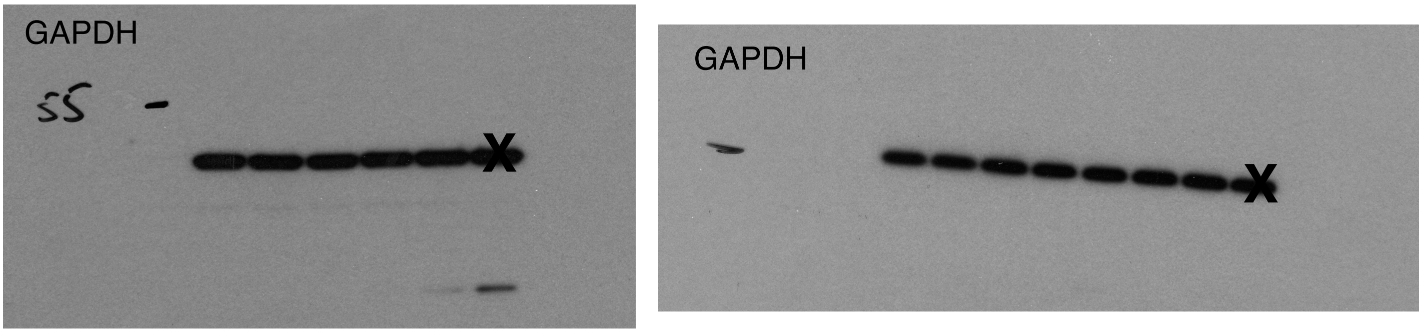

Supplement: Supplementary Information [file srep37775-s1.pdf]
